# Evaluation of *Artemisia amygdalina* D. for Anti-Inflammatory and Immunomodulatory Potential

**DOI:** 10.1155/2013/483646

**Published:** 2013-10-10

**Authors:** Khan Mubashir, Bashir A. Ganai, Khalid Ghazanfar, Seema Akbar, Akhtar H. Malik, Akbar Masood

**Affiliations:** ^1^Department of Biochemistry, University of Kashmir, Srinagar 190006, India; ^2^Regional Research Institute of Unani Medicine, Kashmir University Campus, Srinagar 190006, India; ^3^Centre for Biodiversity and Taxonomy (CBT), Department of Botany, University of Kashmir, Srinagar 190006, India

## Abstract

*Artemisia amygdalina* D. is a critically endangered endemic medicinal plant of Kashmir Himalayas. In the current study anti-inflammatory and immunomodulatory activity of the plant was carried out. Carrageenan paw edema model was used to study the potential of the drug in inflammation in Wistar rats. SRBC-specific haemagglutination titre and DTH assays were carried out in Balb/C mice for observing the effect of test drugs on immune system. The plant extracts used as test drugs showed to have anti-inflammatory potential. The methanolic fraction was observed to have the maximum effect on the inhibition of paw edema formation with the inhibitory potential of 42.26%, while in the immunomodulation studies the test drugs were found to have the immunosuppressant activity with methanolic fraction again showing the maximum potential for the suppression of both humoral (55.89% and 47.91%) and cell-mediated immunity (62.27% and 57.21%). The plant in total seems to have the anti-inflammatory potential. The suppression of immune system suggests some mechanistic way by which the inhibition of inflammation takes place. Since, in chronic inflammation like arthritis, there is the involvement of immune system, the plant in that way may serve as an alternative for the treatment of such autoimmune diseases.

## 1. Introduction

Inflammation is the reaction of living tissues to injury, infection, or irritation. It is an essential protective process preserving the integrity of organisms against physical, chemical, and infective insults. However, it is frequent that the inflammatory response to several insults erroneously leads to the damaging of normal tissues responsible for certain pathological conditions such as heart attacks, septic shocks, and rheumatoid arthritis [1]. One of the early cellular events in inflammation is the migration of leukocytes, primarily neutrophils. This response can be measured by using the neutrophil-specific enzyme myeloperoxidase (MPO), an indicator of neutrophil accumulation [2]. In addition, nitric oxide (NO) and TNF-*α* produced by macrophages play an important role in inflammation, and NO synthase inhibitors can reverse several classic inflammatory symptoms [3]. TNF-*α* is a cytokine which plays an important role in inflammation. TNF-*α* stimulates neutrophils to transcribe and release cytokines and chemokines biosynthesis [4].

In autoimmune diseases, on one hand pathogenic self-reactivity of T cells plays an important role, while on the other hand self-reactivity is needed to regulate autoaggressive responses. Delayed-type hypersensitivity can be elicited in rodents against a variety of antigens such as bacteria, sheep red blood corpuscles (SRBCs), and histocompatibility antigen and is a T-lymphocyte-dependent phenomenon. The arthritogenic T cells likely migrate to the joints and initiate inflammation in the synovium by recruiting other lymphocytes, monocyte/macrophages, and polymorphonuclear leukocytes [5]. These may release cytokines and other products, which contribute to resorption of bone and destruction of cartilage. Thus, pharmacological inhibition of this leukocyte migration and accumulation in arthritis may have beneficial effects for joint preservation [6]. The most challenging question for the study of rheumatoid arthritis concerns the specificity of immune reactions, which indicate and perpetuate the autoimmune pathology. Those reactions are most likely dependent on activated autoreactive T cells but do also involve certain autoreactive B cells and such immune specific lymphocytes can be anticipated to be involved in both delayed-type hypersensitivity (DTH) and immune complex-mediated pathogenic inflammation [7].

*Artemisia amygdalina *D. is an endemic medicinal plant of Kashmir valley belonging to the family Asteraceae and grows in subalpine region of Kashmir Himalaya and North-West Frontier Province of Pakistan [8]. The plant extract is used locally for the treatment of epilepsy, piles, nervous disorders, cough, cold, fever, and pain [9]. The women folk of the valley use it for amenorrhea and dysmenorrhoea [10]. The major active principles in this plant are the terpenes, p-cymene, and 1,8-cineole [11]. As a consequence of overharvest and deforestation, this plant is considered as the critically endangered endemic species of Kashmir valley [8].

The current study was carried out to evaluate the anti-inflammatory potential of this plant and then to study its effect on the immune system.

## 2. Materials and Method

### 2.1. Collection and Identification of Plant Material

The plant material was procured from Kashmir University Botanical Garden (KUBG) and identified in the Centre of Plant Taxonomy (COPT), Department of Botany, University of Kashmir, India. The specimen is retained in the herbarium of COPT vide Voucher no. 1803.

### 2.2. Preparation of Extracts

The whole plant (aerial) was washed, cut into small pieces, and shade-dried. The plant material was pulverized into coarse powder and extracted successively using petroleum ether, ethyl acetate, methanol, and water, respectively, by soxhlet extraction. The solvents were allowed to evaporate in a rotary evaporator at 40°–45°C, and the extracts obtained were stored in a refrigerator at 4°C. The yields of the petroleum ether, ethyl acetate, methanol, and aqueous extracts were 4.8, 5.2, 5.8, and 5.3% (w/w), respectively.

### 2.3. Animals

Male albino Wistar rats (120–140 gm) in groups of 4 each were used for anti-inflammatory study. Drugs were prepared in 1% Tween 20 and administered orally at doses of 250 mg/kg bw. Diclofenac was used as an anti-inflammatory drug at a dose of 20 mg/kg (p.o.).

Male Balb/C mice of 8–10 weeks old and weighing 18–22 g, in groups of five each, were used for the immunomodulatory study. Drugs for oral administration were freshly prepared as a homogenised suspension of different extracts of *A. amygdalina* D. at doses of 100 mg/kg each in 1% Tween 20 and administered orally, once daily for the duration of the experiment to Balb/C mice. Cyclophosphamide was used as the standard immunosuppressive agent at 50 mg/kg (p.o.).

The animals were housed under standard laboratory conditions with a temperature of (25 ± 2)°C, relative humidity of (55 ± 10)%, and 12/12 h light-dark cycles and fed with standard pellet diet (Lipton India Ltd.), and water was given *ad libitum*. None of the animals was sacrificed throughout the study.

### 2.4. Chemicals

Tween 20, cyclophosphamide, and diclofenac were purchased from Sigma Chemical Co. (St. Louis, MO, USA). All other reagents used were of analytical grade.

 Fresh blood was collected from a healthy sheep in the animal house. Sheep red blood cells (SRBCs) were washed thrice with normal saline adjusted to a concentration of 1 ml, containing 5 × 10^9^ cells for immunisation and challenge.

### 2.5. Experimental Protocols

All experimental protocols and the number of animals used for the experimental work were duly approved by the Institutional Animals Ethics Committee (IAEC); of Indian Institute of Integrative Medicine (CSIR), Canal Road Jammu (CPCSEA registration no. 67/CPCSEA/99).

### 2.6. Carrageenan-Induced Paw Edema

Carrageenan-induced paw edema model [12] was utilized to assess the acute anti-inflammatory potential of the test samples. Animals were divided into six groups (*n* = 4). Group I served as control, rats in groups II–V were administered with plant extracts, and group VI was used as positive control. All drugs were given orally 45 min before carrageenan injection. Carrageenan was prepared in normal saline (1%), and 0.1 ml was injected into the subplantar region of left hind paw. The volume of both paws was measured with volume differential meter (520-R, IITC Life Science, USA) after 4 h with the volume of right paw taken as uninjected paw volume. Percent inhibition was calculated by taking mean of the difference of right and left paw oedema, using the formula

(1) $$\begin{array}{c} \%\;\text{inhibition}=\frac{C-T}{C}\times 100, \end{array}$$

where *C* is mean oedema in the control group and *T* is mean oedema in the treated group.

### 2.7. Humoral Antibody Response

The mice were divided into 6 groups, each consisting of 5 animals. Mice in group I (control) were given 1% Tween 20, 0.2 ml/mouse for 14 days. Mice in group II–V were given 100 mg/kg bw (orally) for 14 days. Mice in group VI were given cyclophosphamide 50 mg/kg on day 1 and continued for 14 days. The animals were immunised by injecting 200 *μ*L of 5 × 10^9^ SRBCs/mL, intraperitoneally (i.p.) on day 1. Blood samples were collected in microliter tubes from individual animals of all the groups by retro-orbital vein puncture on day 7 and day 14. The blood samples were centrifuged, and the serum was separated. Then, haemagglutination primary and secondary titres were performed [13, 14].

### 2.8. Delayed-Type Hypersensitivity

A new area of research is the discovery or/and development of immunomodulatory agents that are free from any toxic side effects and can be used for a long duration, resulting in continuous immunoactivation/suppression [15]. Animals were divided into six groups of 5 each. Group I served as sensitized control, as in humoral antibody titre. Mice in group II–V were administered extracts of *A. amygdalina* after SRBCs' sensitization and once daily for seven days. Cyclophosphamide (50 mg/kg) was administered as standard T-cell suppressor (group VI). The mice were challenged by injecting the same amount of SRBCs intradermally into the right hind footpad, whereas left hind footpad served as control [16, 17].

The footpad thickness was measured with sphaeromicrometer (pitch 0.01 mm) at 24 and 48 h of SRBCs' challenge.

## 3. Results and Discussion

### 3.1. Carrageenan-Induced Paw Edema

Inflammation is a protective process that is essential for the preservation of the integrity of the organism in the event of physical, chemical, and infectious damages. Often, the inflammatory response to severe lesions, erroneously damages normal tissue [18]. The injection of carrageenan into the hind paw of rats elicits an acute inflammatory response characterized by accumulation of fluid (edema). During acute inflammation, serum proteins and leukocytes migrate to areas of tissue injury. Recruitment of cells to inflammatory sites is dependent on the release of vasoactive and chemotactic factors that increase regional blood flow and microvascular permeability and promote the migration of leukocytes from the intravascular space into the tissues [19]. The different extracts tested orally for anti-inflammatory activity at a dose of 250 mg/kg showed decrease in the paw edema after 4 h. Methanolic extract showed maximum inhibition in paw edema (45.26%) as compared to control followed by aqueous (29.47%), ethyl acetate (18.95%), and petroleum ether (3.16%) extracts (Table 1). The inhibition in the paw edema of standard group observed was 52.63%. The % inhibition by methanolic and standard drug is very much comparable and is statistically significant at *P* < 0.05, while the results obtained in the petroleum ether-treated group are almost same as that of the control group.

### 3.2. Humoral Antibody Titre

**Figure 1:**
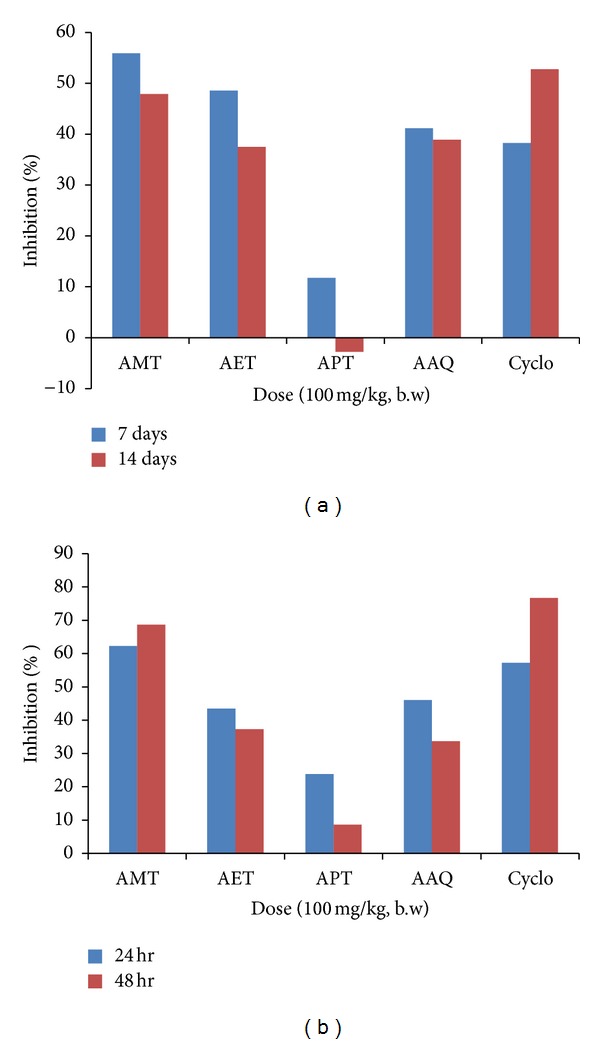
(a) Graph showing % age inhibition of primary and secondary response in Balb/C mice by different fractions of *A. amygdalina* D. (b) Graph showing 24 h and 48 h % age inhibition of cell-mediated response in mice by different fractions of *A. amygdalina* D.

The antibody titre was determined to establish the humoral response against sheep RBC. At neutral pH, red blood cells possess negative ions cloud that makes the cells repel from one another; this repulsive force is referred to as zeta potential. Because of its size and pentameric nature, IgM can overcome the electric barrier and get cross-link red blood cells, leading to subsequent agglutination [20]. The different fractions of *A. amygdalina *used produced decreased the effect on primary and secondary antibody formation compared to control. But methanolic fraction produced maximum decrease in humoral response followed by ethyl acetate and aqueous fractions at a dose of 100 mg/kg (Table 2). The observed decrease in primary and secondary antibody titres in methanolic fraction is 55.89% and 47.91%, ethyl acetate fraction is 48.53% and 37.5%, and aqueous fraction is 41.17% and 38.89%, respectively. Petroleum ether fraction showed decrease in primary response (11.76%) but a slight increase in secondary response (2.77%). The immunosuppressant activity shown by the three fractions is very much comparable to cyclophosphamide 50 mg/kg used as a standard drug inducing 38.23% and 52.77%, decrease in primary and secondary titres, thus indicating that these fractions of *A. amygdalina* significantly (*P* < 0.05) inhibit antibody formation (Figure 1(a)). The production of primary antibodies was more pronounced as compared to secondary antibodies.

### 3.3. Delayed-Type Hypersensitivity Response

DTH reaction is characterised by large influxes of nonspecific inflammatory cells, in which the macrophage is a major component. It is a type IV hypersensitivity reaction that develops when antigen activates sensitized T cells. Activation of T cells by antigen presented through appropriate antigen-presenting cells results in the secretion of various cytokines including interleukin-2, interferon-*γ*, macrophage migration inhibition factor, and tumor necrosis factor-*β* [21]. The T-cell-mediated DTH response to sheep RBC showed a decrease in paw volume in test groups as compared to control group. Out of the different test groups, methanolic fraction-treated group showed maximum decrease in paw volume which is very much comparable to the results seen in cyclophosphamide-treated group used as standard drug (Table 3). The percentage decrease in edema formation in methanolic fraction-treated group and standard group observed is 62.27% and 68.65% and 57.21% and 76.71% after 24 and 48 h, respectively (Figure 1(b)). The results observed in test groups were very much significant (*P* < 0.05) compared to the control group.

## 4. Conclusion

Advances made in recent years in order to understand the cellular and molecular bases of immune response and the identification of small peptides capable of regulating this process gave us understanding to alter the immune response in favour of healthy state [22]. The therapeutic efficiency of some herbs may, in part, be mediated via their influence on the immune response, since some of these plants can affect the immune reactions through their anti-inflammatory actions [23]. Similar type of results was observed in the current study of *A. amygdalina*. The plant showed anti-inflammatory and also immunosuppressive activity. Based on the above results and analysis, it can be concluded that *A. amygdalina* has the potential to suppress cell-mediated immunity as well as humoral immunity, and it may be a potential therapeutic candidate in several immunostimulant clinical conditions. From the current study, it can be said that this plant may be a good resource of bioactive components especially flavonoids which have been found to have the immunomodulatory and anti-inflammatory activity [24]. Further detailed studies on its mode of immune action and active constituent's isolation are in progress.

## Figures and Tables

**Table 1:** Effect of different extracts of *Artemisia amygdalina* D. on Carrageenin-induced paw edema in rats (mean ± SE) (*n* = 4).

S. no.	Groups	Dose (mg/kg)	Initial paw vol. (mL)	Paw vol. after 4 h (mL)	Edema (4 h)	% age inhibition (4 h)
1	Control	NS	0.95 ± 0.03	1.9 ± 0.04	0.95 ± 0.03^a^	—
2	AMT	250	1 ± 0.00	1.52 ± 0.05	0.52 ± 0.05^b^	45.26
3	AET	250	0.97 ± 0.05	1.75 ± 0.06	0.77 ± 0.05^b^	18.95
4	APT	250	0.95 ± 0.04	1.87 ± 0.09	0.92 ± 0.07^ac^	3.16
5	AAQ	250	0.92 ± 0.02	1.6 ± 0.04	0.67 ± 0.05^bc^	29.47
6	Diclo	20	1.07 ± 0.07	1.52 ± 0.05	0.45 ± 0.03^c^	52.63

Values along the same column with different superscripts are statistically significant to each other using Tukey's HSD test (*P* < 0.05). AMT: methanolic extract; AET: ethyl acetate extract; APT: petroleum ether extract; AAQ: aqueous extract; Diclo: diclofenac.

**Table 2:** Effect of different extracts of *A. amygdalina* D. on haemagglutination titre in mice (mean ± SE) (*n* = 5).

Humoral response			
Groups	Dose (mg/kg)	Primary titre	Secondary titre

Control	SRBC	6.8 ± 0.38^a^	7.20 ± 0.49^a^
Methanolic fraction	100	3.0 ± 0.41^c^	3.75 ± 0.43^b^
Ethyl acetate fraction	100	3.5 ± 0.29^c^	4.50 ± 0.26^b^
Petroleum ether fraction	100	6.0 ± 0.45^ab^	7.40 ± 0.24^a^
Aqueous fraction	100	4.0 ± 0.89^bc^	4.40 ± 0.24^b^
Cyclophosphamide	50	4.2 ± 0.58^bc^	3.40 ± 0.24^b^

Values along the same column with different superscripts are statistically significant to each other using Tukey's HSD test (*P* < 0.05).

**Table 3:** Effect of different extracts of *Artemisia amygdalina* D. on delayed-type hypersensitivity response in mice (mean ± SE) (*n* = 5).

DTH assay			
Groups	Dose (mg/kg)	24 hr paw thickness (mm)	48 hr paw thickness (mm)

Control	SRBC	0.6825 ± 0.04^a^	0.335 ± 0.021^a^
Methanolic fraction	100	0.2575 ± 0.02^c^	0.105 ± 0.012^d^
Ethyl acetate fraction	100	0.386 ± 0.04^bc^	0.21 ± 0.023^c^
Petroleum ether fraction	100	0.52 ± 0.04^b^	0.306 ± 0.021^b^
Aqueous fraction	100	0.368 ± 0.032^c^	0.222 ± 0.027^c^
Cyclophosphamide	50	0.292 ± 0.032^c^	0.078 ± 0.006^d^

Values along the same column with different superscripts are statistically significant to each other using Tukey's HSD test (*P* < 0.05).
